# 

**Published:** 1910-07

**Authors:** 


### INDEX.

Abstracts and Selections......25, 53, 98, 152, 174, 226, 267, 298, 346 ........................................ 420
A Department of Public Health........................ 14
Appreciation ........................................ 297
A Question of Rights and Privileges. By George L. Servoss, M. D., Fairview, Nevada........................... 330
Asthma. By William Francis Waugh, A. M., M. D., Dean of Bennett Medical College, Chicago, Ill............. 7
Atropine. By George L. Servoss, M. D., Fallon, Nevada.... 405
Books and Magazines......72, 188, 232, 268, 310, 354, 389, 434
Bryonin. By H. H. Redfield, M. D., Chicago............ 409
Collinsonin. By H. H. Redfield, M. D., Chicago, Ill....... 286
Correspondence ..................................97, 157
Diphtheria. By William F. Waugh, M. D., Chicago........ 335
Diuretics. By George L. Servoss, M. D., Fairview, Nevada.. 204
Editorial Department........19, 51, 93, 140, 169, 217, 261, 291 339, 381, 414........................................ 463
Editorialets........20, 51, 96, 147, 170, 222, 265, 343, 384, 469
Emergency Remedies. By George L. Servoss M. D., Fairview, Nevada ...................................... 39
How Inebriety Might Be Prevented by Early Education. By Tom A. Williams, M. B., C. M., Edin., Washington, D. C. 122
Immortality. F. E. Daniel............................ 462
Individuality in Evolution. By John C. Warbrick, M. D., Chicago ............................................ 327
Injuring Business ...................................... 19
Iodide of Potassium in Tertiary Syphilis. By S. O. Young, M. D., Galveston, Texas........................... 163
Louisiana National Life Assurance Society, New Orleans.... 363
More on Vasectomy. By Dr. G. Henri Bogart, Brookville, Indiana ............................................ 239
Municipal Hospital Care of Contagious Diseases. By A. W. Fly, M. D., Galveston, Texas....................... 157
Publisher's Department......30, 74, 110, 154, 190, 236, 271, 312, 356, 396, 437................................ 478
Quinine Amaurosis, with Report of Three Cases. By B. H. Booth, M. D., Drew, Mississippi.................... 46
Quinine Amaurosis—Report of Two Additional Cases. By B. H. Booth, M. D., Drew, Miss.................... 412
Rapid Bloodless Circumcision and Its Technic by Use of a New Instrument. By S. L. Kistler, Los Angeles, Cal.. 445
Reports of Two Cases of Taenia Saginata. By H. D. Eaton, M. D., Chihuahua, Mexico...........................200

Reversion of Types. By S. O. Young, M. D., Galveston, Texas .... 47
Sanitarium Bells. By Mrs. W. J. Harvey, Denison, Texas.. 287
Society .... 208
Sterilization—"The Indiana Plan." By Dr. G. Henri Bogart, Brookville, Indiana.... 79
Sterilization of the Unfit. By G. Henri Bogart, M. D., Brookville, Indiana .... 197
Sterilization of the Unfit. By Dr. G. Henri Bogart, Brookville, Indiana .... 279
Suggestion as a Factor in Causing Neuroses and Psychoses. By W. T. Marrs, M. D., Peoria Heights, Ill.... 336
Symptoms and Conditions Following the Subsidence of the Alcoholic Disease. By T. D. Crothers, M. D., Hartford, Conn .... 321
Syphilis. By William Francis Waugh, A. M., M. D., Chicago, Ill.... 165
The Alcoholic Case and a Surgical Operation for the Cure of Chronic Alcoholism. By J. W. Kenney, M. D., San Antonio Texas .... 117
The Burden of Prudery. By Dr. G. Henri Bogart, Brookville, Indiana .... 328
The Evolution of Medicine and Surgery for the Past Thirty-five Years. By A. W. Fly, M. D., Galveston, Texas.... 1
The Hookworm, the Antidote to Effort; the Germ of *Dolce Far Niente,* the South's "Bromide." By a Southern New Yorker .... 448
The Lying Patent Medicine Man and the False Certifier. By Charles H. Hughes, M. D., St. Louis, Mo.... 452
"The Social Evil." By Winslow Anderson, M. D.... 367
The Sources of Hookworm Infection and the Manner in Which Infection Takes Place. By C. C. Bass, M. D., New Orleans, La.... 132
The Treatment of Chronic Eczema of Children. By Dr. Wenzel, St. Joseph, Mo.... 451
To Our Wives and Sweethearts—A Toast.... 12
Treatment of Tuberculous Glands. By Emory Lanphear, M. D., St. Louis, Ill.... 257
Treatment of Wounds or Surgical Dressings. By Dr. L. Sexton, Tulane, New Orleans.... 251
What is the Physician's Duty in the Prevention of Moral and Social Diseases? By F. W. Sears, M. D., Syracuse, New York .... 373

